# A late IL-33 response after exposure to *Schistosoma haematobium* antigen is associated with an up-regulation of IL-13 in human eosinophils

**DOI:** 10.1111/pim.12035

**Published:** 2013-07-01

**Authors:** S Wilson, F M Jones, H K M Fofana, A Landouré, G Kimani, J K Mwatha, M Sacko, B J Vennervald, D W Dunne

**Affiliations:** 1 Department of Pathology, University of CambridgeCambridge, UK; 2 Institut National de Recherche en Santé PubliqueBamako, Mali; 3 Kenya Medical Research InstituteNairobi, Kenya; 4 DBL – Centre for Health Research and Development, Faculty of Life Sciences, University of CopenhagenCopenhagen, Denmark

**Keywords:** eosinophils, helminth, human, IL-13, IL-33, schistosomiasis, ST2

## Abstract

IL-33, a proposed alarmin, stimulates innate immune cells and Th2 cells to produce IL-13 and is rapidly upregulated upon antigen exposure in murine helminth infection. The human IL-33 response to helminth antigen was analysed in Malians infected with *Schistosoma haematobium* by disrupting parasite integrity via chemotherapy. Plasma IL-33 was measured pretreatment, and 24 h and 9 weeks post-treatment. At 24 h post-treatment, IL-33 levels were low. Nine week post-treatment IL-33 levels were elevated and were associated with an increase in intracellular IL-13 in eosinophils. Up-regulation of intracellular IL-13 in eosinophils was also associated with eosinophil expression of ST2L, the IL-33 receptor. IL-33 may play an important downstream role in the human response to schistosome adult worm antigen exposure.

## Introduction

IL-33, a member of the IL-1 family with a reported nuclear location, is hypothesized to be an ‘alarmin’ released by tissue cells, primarily epithelial, endothelial and smooth muscle cells, in response to necrotic damage (1,2). IL-33 signals through transmembrane ST2L (1) and coreceptor, IL-1R accessory protein (3). ST2L expression is restricted to mast cells (4), basophils (5), eosinophils (6), type 2 innate lymphoid cells (ILC2) or nuocytes (7,8), and dendritic (9) and Th2 cells (10). Thus, IL-33 signalling is restricted to type 2 immune cells – cells majorly associated with allergy and helminth infection. A soluble splice variant of ST2 (sST2) acts as a decoy receptor, regulating the IL-33/ST2L axis (11).

In murine gut helminth infection, early IL-33 release activates ILC2 to produce IL-13 (7). IL-13 can drive non-IgE-dependent expulsion of gut nematodes, via goblet cell hyperplasia (12), altered mucin production (13) and smooth muscle contraction (14). Eosinophilia is another hallmark of helminth infection, and for schistosomes, partial human immunity to re-infection is eosinophil dependent (15). Following treatment of human schistosome infection with praziquantel, which disrupts the integrity of adult worms exposing previously cryptic antigens, an increase in eosinophil number is observed (16). This *in vivo* exposure to antigen also stimulates early release of type 2-associated cytokines, with plasma IL-5 in particular being elevated 24 h post-treatment (17). To determine whether IL-33 plays a role in the human response to schistosome-derived antigens, levels of IL-33 post-treatment for *S. haematobium* were examined at the early time point of 24 h, when circulating IL-5 level is elevated, and at the later time point of 9 weeks post-treatment, when the IL-5 circulating levels have returned to pretreatment levels.

## Materials and Methods

### Study population and sample collection

Twenty-eight females and 19 males, aged 5 to 39 years (mean = 14·93 years) from Segou Region, Mali, participated in the study. For analysis, age was split into two groups: 5–11 years (*n* = 25) and >11 years (*n* = 22). The participants were a subcohort of a wider community-based study comparing the effects of receiving one or two praziquantel treatments. Twenty-seven participants were from the village of Kalabougou and 20 participants from the villages of Guenidaga and Kaladangan. The study population and site is described in more detail elsewhere (18). Subcohort inclusion was based on having detectable *S. haematobium* infection*,* but no detectable *S. mansoni* or gut helminth infections and receiving one dose of praziquantel. Quantitative parasitology was carried out on three urine samples collected pretreatment and 9 week post-treatment. Pretreatment, a median infection intensity of 36 eggs/10 mL (interquartile range, 7·25, 102) was detected. Twenty-one individuals had a high infection intensity of >50 eggs/10 mL of urine. Treatment was 82·61% effective, with only eight individuals having detectable infections at 9 week post-treatment; one parasitological count was missing at this time point. Amongst those still with detectable infections, the median intensity was one egg/10 mL (interquartile range, 1, 2·25).

Five millilitre of blood was collected into EDTA pretreatment and 9 weeks post-treatment. Two hundred microlitres of blood was removed for haematology analysis, including eosinophil counts, and preparation of malaria parasitaemia slides. Platelet-poor plasma was harvested by centrifugation and treated with 0·3% tributyl phosphate/1% Tween 80 (Sigma, Poole, UK) to inactivate encapsulated viruses. The cell pellet from centrifugation was retained, and red blood cells were lysed in 0·15 m NH_4_Cl /0·01M KHCO_3_/5 mm EDTA. The resulting white blood cell pellet was washed in PBS/2% FCS/5 mm EDTA, fixed in 2% PFA for 6 min at 37°C and cryopreserved in FCS/10% DMSO. Three finger-prick samples, one pretreatment, one 24 h and one 9 weeks post-treatment, were collected into EDTA and the plasma was harvested. The Ethical Review Committee of the National Institute for Research in Public Health, Mali, approved the study. Informed consent was given by participating adults and parents/guardians of participating children.

### Cytokine assays and flow cytometry

IL-5, IL-13 and IL-33 levels were measured in finger-prick plasma samples by Luminex bead array. Beads were coupled with capture monoclonal Ab (IL-5 and IL-13, BD Pharmingen, San Diego, CA, USA; IL-33, R&D Systems, Minneapolis, MN, USA), incubated with 12·5 μL plasma, diluted 1 : 8, overnight at 4°C, and levels were detected using monoclonal Ab for IL-5 and IL-13 (BD Pharmingen), and polyclonal goat anti-human IL-33 (R&D Systems). Detection limits were 1·2 pg/mL for IL-5 and IL-13, and 3·88 pg/mL for IL-33. sST2 was measured by ELISA in venous plasma samples using a matched antibody pair (R&D Systems). Plates were coated with 1 μg/mL capture Ab, samples were incubated overnight at 4°C, and sST2 was detected with 0·1 μg/mL detecting Ab. The assay had a detection limit of 22 pg/mL. Assay detection limits were assigned to samples in which levels were undetectable.

Cells were snap-thawed at 37°C, washed in PBS/5% FCS, incubated in Fc block (Miltenyi Biotec, Cologne) and surfaced stained with anti-human ST2L (MBL, Woburn, MA, USA), diluted in PBS/5% FCS, on ice for 1 h, then washed, surface staining fixed and stained for intracellular IL-5 (Pharmingen) and IL-13 (R&D systems) for 30-min at room temperature in HBSS/0·1% saponin. Flow cytometry data were acquired on a Cyan ADP MLE. Eosinophils from *S. haematobium-*infected individuals are very granular and are easily differentiated from neutrophils on forward scatter/log side scatter profiles after saponin treatment. Eosinophil gating was confirmed by their autofluorescence properties. The percentage of ST2L surface expression was determined using fluorescence minus one-gating, and eosinophil intracellular IL-5 and IL-13 were analysed as median fluorescence intensity (MFI). Gating strategy is shown in Figure S1. For each individual and time point, an unstained sample was run as an autofluorescence control.

### Statistical analysis

As data were skewed, initial analysis was conducted by nonparametric tests. Mann–Whitney *U*-tests were used to compare the levels of IL-33 between groups of individuals. Paired longitudinal data were analysed using Wilcoxon tests. Linear regression models were constructed for intracellular IL-13 and IL-5. IL-33 and sST2 levels at 9 weeks post-treatment were log-transformed prior to being entered into models. Models were adjusted for autofluorescence by inclusion of MFI of unstained cells in the appropriate fluorochrome channel. Models were compared by anova.

## Results and Discussion

**Figure 1:**
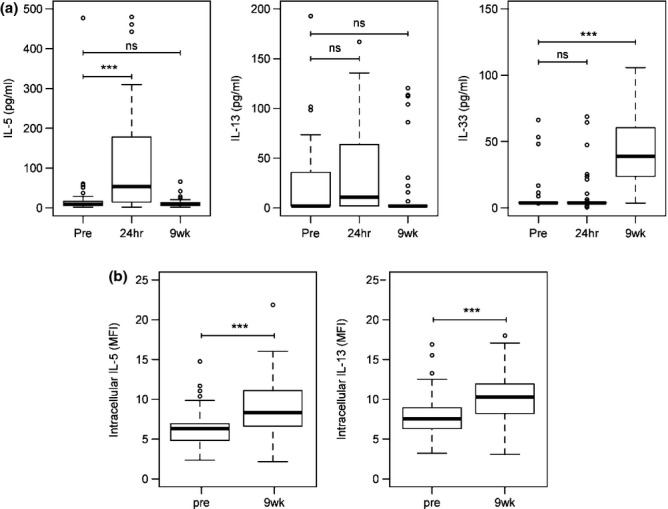
Circulating levels of IL-5, IL-13 and IL-33 and eosinophil intracellular IL-5 and IL-13. (a) Shown are boxplots, representing the cohort's median and interquartile ranges, of the circulating levels of IL-5, IL-13 and IL-33 measured pretreatment, 24 h and 9 weeks post-treatment. ***P <* *0·001, ns, not significant by Wilcoxon test. (b) Shown are boxplots, representing the cohort's median and interquartile ranges, of the median fluorescence intensity of intracellular IL-5 and IL-13 in eosinophils collected pretreatment and 9 weeks post-treatment. ***P <* *0·001 by Wilcoxon test.

For this cohort, circulating levels of IL-5 at 24 h post-treatment were higher compared with pretreatment levels (Figure1a; *U* = 30·5, *P *<* *0·001), but IL-13 levels were not (*U* = 222, *P *=* *0·083). Circulating levels of IL-5 and IL-13 9 weeks post-treatment were not significantly different from pretreatment levels (*U* = 664, *P *=* *0·292 and *U* = 204, *P *=* *0·126, respectively). A boost in circulating IL-5, but not IL-13, levels 24 h post-treatment is in concurrence with a previous study of *S. mansoni-*infected Ugandan fishermen (17). Levels of IL-33 were low pre- and 24 h post-treatment, being detectable for only eight individuals (17·02%) at both time points. At 9 weeks post-treatment, levels of IL-33 were significantly higher than at pretreatment (*U* = 100, *P *<* *0·001; Figure1a). IL-33 levels 9 weeks post-treatment were not significantly associated with sex, age, village of residence or heavy pretreatment infection intensity (data not shown). A nonsignificant rise in prevalence of malaria parasitaemia from 72 to 88% occurred (χ^2^ = 0·0427, *P *=* *0·8362), and 9 week IL-33 levels were not significantly associated with having detectable parasitaemia at this time point (*U* = 149, *P *=* *0·4157).

The elevation of IL-33 at 9 weeks post-treatment but not 24 h post-treatment does not preclude an early role for IL-33 in response to *S. haematobium* antigen exposure, upstream of the IL-5 boost. In atopic dermatitis patients, exposed to house dust mite skin patches, IL-33 mRNA up-regulation peaks 6 h post-allergen exposure (19). A similar up-regulation in IL-33 mRNA could have occurred in this study. However, protein levels of IL-33 were not measured in the atopic dermatitis study, and IL-33 mRNA expression was not assessed in the current study, so how mRNA and protein levels correspond is not known, a pertinent point, when the poor understanding of the mechanisms involved in processing IL-33 from its pro- to mature form (20), and its release from cells (21) is taken into account. The lack of IL-33 protein detected at 24 h post-treatment may be indicative of the rapid type 2 cytokine responses seen after *S. haematobium* treatment being IL-33 independent.

The boost in IL-33 at 9 weeks post-treatment does not necessarily concur with its proposed role as an alarmin. It has recently been shown that IL-33 release is not dependent on necrosis, as it can be trafficked from the nuclear region, via the pore complex, to cytoplasmic granules and released by living cells, although under biomechanical strain (21). The release of IL-33 via cytoplasmic granules may occur in the late response post-treatment. Alternatively, low levels of IL-33 transcription have been reported in human dendritic cells and macrophages (1), and up-regulation of IL-33 levels by these cells could be the source of the elevated IL-33 observed.

ST2L is expressed in human eosinophils (22). Here, although individuals showed changes in percentage of eosinophils with detectable ST2L expression pre- to 9 weeks post-treatment (Figure S2a), with 15 of 47 (31·9%) having a >10% decrease and 15 of 47 (31·9%) having a >10% increase, there was no significant difference in ST2L expression at cohort level (*U* = 584, *P *=* *0·838). Levels of sST2 also fluctuated at the individual level (Figure S2b), with 14 individuals (29·8%) having a decrease of >40 pg/mL pre- to 9 weeks post-treatment and 14 others having a > 40 pg/mL increase. Again there was no significant difference at cohort level (*U* = 430, *P *=* *0·608).

Human eosinophils are able to make IL-13 (23), and IL-33 could increase IL-13 production by these cells, similar to its ability to increase IL-13 production by murine eosinophils (24,25). Significant increases in levels of eosinophil IL-13 MFI (*U* = 192, *P *<* *0·001) were observed 9 weeks post-treatment (Figure1b). In linear regression models, IL-33 levels and the percentage of eosinophils with detectable ST2L expression at 9 weeks post-treatment were significantly associated with the MFI of eosinophil intracellular IL-13 (Table1). The addition of sST2 levels at nine weeks significantly improved the model (*F* = 4·581, *P *=* *0·038). The inclusion of sST2, so controlling for levels of this regulator, also increased the strength of the relationship between intracellular IL-13 and IL-33 [from β = 0·103 (SE = 0·043)] and ST2L expression [from β = 0·007 (SE = 0·002)]. When pretreatment levels of ST2L or sST2 were entered, neither were significant (data not shown); so although at cohort level no significant difference was observed in ST2L and sST2 pre- to post-treatment, individual levels were important in their relationship with IL-13 production by eosinophils. The strengthening of the relationship between eosinophil intracellular IL-13 production and plasma IL-33 and its ligand when plasma sST2 was added to the model suggests that the relationship is under regulation by the antagonistic soluble receptor for IL-33. The positive association between sST2 and intracellular IL-13 MFI probably reflects the increased need for regulation of the IL-33/ST2L axis, as IL-13 production increases.

**Table 1:** Linear regression model of eosinophil intracellular IL-13 at 9 weeks post-treatment

	β (SE)	*P*
Log IL-33	0·128 (0·042)	0·003
ST2L expression (%)	0·008 (0·003)	0·005
Log sST2	0·110 (0·046)	0·021

Model was adjusted for unstained eosinophil autofluorescence.

The IL-33 may be playing a delayed role in tissue repair. In humans, intradermal allergen challenge of atopic volunteers leads to infiltrating eosinophils, and IL-13 from eosinophils upregulates the production of extracellular protein by fibroblasts (26). In murine skin, IL-33 induces fibrosis, a process dependent upon eosinophilic infiltration and production of IL-13 (24). Atopic dermatitis patients also have a high number of ILC2 in skin lesions (27), a cell type has been shown to induce expulsion of murine gut helminthes via IL-13 production (7). In murine models of atopic dermatitis, although ILC2 produces IL-13, this is independent of IL-33 (27). If the IL-33 is elevated because of a tissue repair response post-treatment for *S. haematobium*, it may be acting through eosinophils rather than through ILC2. However, the low levels of IL-13 at 9 weeks post-treatment suggest that the eosinophils may make IL-13 but not release it.

IL-33 has also been reported to delay apoptosis of eosinophils (22), a cell type associated with immunity to schistosomiasis (15); however, no significant difference was observed in eosinophil number pre to 9 week post-treatment in this cohort (*U* = 437, *P *=* *0·260). The role of the increased IL-13 by eosinophils therefore remains to be determined, although IL-13 can drive antibody class switching to IgE, and levels of IgE specific to antigen derived from adult schistosome worms are elevated 9 weeks post-treatment (28).

Intracellular IL-5 MFI was also significantly higher at 9 weeks post-treatment (*U* = 192, *P *<* *0·001, Figure  1b). In regression models, there was no significant relationship between IL-5 MFI and 9 week post-treatment IL-33 levels (β = 0·075 (SE =0·062), *P *=* *0·230). Whether this is due to the increase in IL-5 production by eosinophils being independent of IL-33 or whether it was due to lack of statistical power cannot be determined.

To conclude, after chemotherapy-induced *in vivo* exposure to schistosome antigen, IL-33 release was late, being detectable at 9 weeks but, with the exception of a few individuals, not at 24 h post-treatment. The IL-33 released was significantly associated with post-treatment changes in eosinophil IL-13 production, at a time when the circulating levels of rapidly released cytokines, such as IL-5, have decreased. These results indicate that late IL-33 production was playing a downstream role after exposure to schistosome worm antigen. Whether this IL-33 response is associated with partial immunity to re-infection or wound repair, after antigen exposure–induced tissue inflammation, remains to be clarified, and further studies are required to further examine the kinetics of IL-33 production after treatment of *S. haematobium*.

## Supporting Information

**Figure S1.** Eosinophil gating. **Figure S2.** Eosinophil expression of ST2L and circulating levels of sST2.
