# Supplementary material for: A late IL-33 response after exposure to Schistosoma haematobium antigen is associated with an up-regulation of IL-13 in human eosinophils
Source: Parasite Immunol. 2013 Jul 1;35(7-8):224–8. doi: 10.1111/pim.12035 (PMC4463766; doi:10.1111/pim.12035)
Supplement: Supplementary file 1 — Figure S1. Eosinophil gating. Figure S2. Eosinophil expression of ST2L and circulating levels of sST2. [file pim0035-0224-sd1.docx]

**Late IL-33 Response After *In Vivo* Exposure to *Schistosoma haematobium* Antigen is Associated with an Up-regulation of IL-13 in Human Eosinophils**

Shona Wilson*^1^, Frances M Jones^1^, Hassan KM Fofana^2^, Aly Landouré^2^, Gachuhi Kimani^3^, Joseph K Mwatha^3^, Moussa Sacko^2^, Birgitte J Vennervald^4^, David W Dunne^1^

^1^Department of Pathology, University of Cambridge, Tennis Court Road, Cambridge, CB2 1QP, UK

^2^Institut National de Recherche en Santé Publique, Bamako, Mali

**^3^**Kenya Medical Research Institute, Nairobi, Kenya

**^4^**DBL – Centre for Health Research and Development, Faculty of Life Sciences, University of Copenhagen, Thorvaldsensvej 57, 1870 Frederiksberg C, Denmark

*Corresponding author. Tel: +44 1223 33338 E-mail: [sw320@cam.ac.uk](mailto:sw320@cam.ac.uk)

**Figure S1. Eosinophil gating**


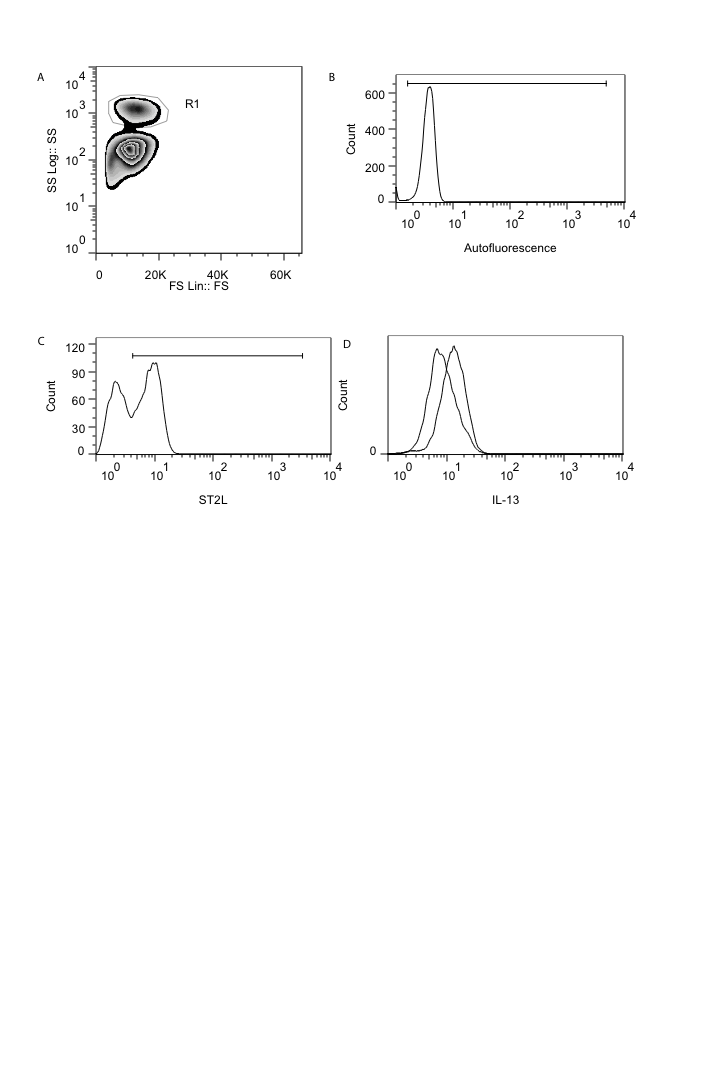


A) Shown is an individual example of forward scatter/log side scatter profile, with eosinophils (region 1) being easily differentiated from neutrophils after saponin treatment. B) Eosinophils were further gated in an unstained channel on their auto-fluorescence properties, excluding cells with low auto-fluorescence that remain after region 1 gating. C) ST2L gating was determined by fluorescence minus one gating. D) Shown is the shift in distribution of intracellular IL-13 of one individual pre to 9wk post-treatment. Intracellular cytokine staining of IL-5 and IL-13 had continuous distributions and where analysed as median fluorescence intensity.**Figure S2. Eosinophil expression of ST2L and circulating levels of sST2**

**
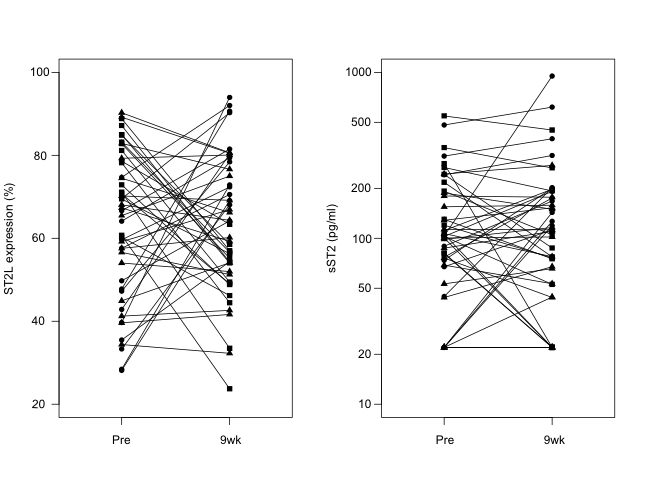
**

A) B)

A) Shown are the percentage of eosinophils expressing detectable levels of ST2L pre-treatment and post-treatment for each individual. Squares >10% decrease in ST2L expression, triangles <10% change in ST2L expression, circles >10% increase in ST2L expression. B) Shown are the levels of circulating sST2 pre-treatment and post-treatment for each individual. Squares >40pg/ml decrease in sST2 level, triangles <40pg/ml change in sST2 level, circles >40pg/ml increase in sST2 level.
